# Dealing with the health state ‘dead’ when using discrete choice experiments to obtain values for EQ-5D-5L heath states

**DOI:** 10.1007/s10198-013-0511-2

**Published:** 2013-07-31

**Authors:** Juan Manuel Ramos-Goñi, Oliver Rivero-Arias, María Errea, Elly A. Stolk, Michael Herdman, Juan Manuel Cabasés

**Affiliations:** 1 HTA Unit of Canary Islands Health Service, Servicio Canario de la Salud, C/Calvario, 271-B, 1 IQZ, 38350 Tacoronte, Santa Cruz de Tenerife, Canary Islands Spain; 2 Red de Investigación de Servicios Sanitarios En Cronicidad (REDISSEC), Servicio Canario de la Salud, Tacoronte, Santa Cruz de Tenerife, Canary Islands Spain; 3 University of Oxford, Oxford, UK; 4 Universidad Pública de Navarra, Navarra, Spain; 5 Institute for Health Policy and Management, Erasmus University of Rotterdam, Rotterdam, The Netherlands; 6 Insight Consulting and Research, Barcelona, Spain

**Keywords:** Discrete choice methodology, Time trade-off, Health state ‘dead’, EQ-5D-5L, EuroQol Group, I19

## Abstract

**Objective:**

To evaluate two different methods to obtain a dead (0)—full health (1) scale for EQ-5D-5L valuation studies when using discrete choice (DC) modeling.

**Method:**

The study was carried out among 400 respondents from Barcelona who were representative of the Spanish population in terms of age, sex, and level of education. The DC design included 50 pairs of health states in five blocks. Participants were forced to choose between two EQ-5D-5L states (A and B). Two extra questions concerned whether A and B were considered worse than dead. Each participant performed ten choice exercises. In addition, values were collected using lead-time trade-off (lead-time TTO), for which 100 states in ten blocks were selected. Each participant performed five lead-time TTO exercises. These consisted of DC models offering the health state ‘dead’ as one of the choices—for which all participants’ responses were used (DC_dead_)—and a model that included only the responses of participants who chose at least one state as worse than dead (WTD) (DC_WTD_). The study also estimated DC models rescaled with lead-time TTO data and a lead-time TTO linear model.

**Results:**

The DC_dead_ and DC_WTD_ models produced relatively similar results, although the coefficients in the DC_dead_ model were slightly lower. The DC model rescaled with lead-time TTO data produced higher utility decrements. Lead-time TTO produced the highest utility decrements.

**Conclusions:**

The incorporation of the state ‘dead’ in the DC models produces results in concordance with DC models that do not include ‘dead’.

## Introduction

The EQ-5D is one of the most widely used preference-based instruments. In 2009, the EuroQol Group released a new version (EQ-5D-5L) of the instrument that included five levels of severity in each dimension, as opposed to three in the original version [1]. For the new instrument to generate a set of societal values for the 3,125 health states, it had to distinguish five levels of severity in five dimensions.

Previous valuation studies had predominantly used time trade-off (TTO) to obtain social preferences from which value sets for EQ-5D health states could be modeled [2–5]. However, increasing the number of health states from 243 to 3,125 made it considerably more costly and complicated to conduct valuation studies based on an interview method such as TTO. Conventional TTO also has problems with health states worse than the state ‘dead’ [6]. These issues led the EuroQol Group to explore new approaches to obtain social values for health states, notably discrete choice (DC) methodology.

In a typical DC task, respondents compare two different options (paired comparison) and indicate which one they prefer. Discrete choice experiments (DCE) have been used extensively in areas such as marketing and transport but not so much in health economics. The use of DCE for health-state valuation is a relatively recent development. Potential advantages include the relative ease of comprehension and administration of ordinal tasks and its greater reliability. DC models may also avoid some of the biases associated with traditional valuation methods [7]. Stolk et al. [8] demonstrated that DC modeling with the classic EQ-5D (three-level) instrument produces values that are congruent with values obtained by other valuation techniques, TTO in particular. That result confirmed previously published findings [9–12].

A question that arises about the use of DC for health-state valuation concerns how to anchor the values produced by the choice model onto the dead (0)—full health (1) scale that is required to compute quality-adjusted life years. One strategy is to use DC data in combination with TTO data. This would entail deriving values from DC data and then using values from TTO to rescale those DC values. The need to collect TTO data alongside a DC study, however, might make the valuation study more complex than necessary. So, instead, the DC task could be designed in such a way that a value for ‘dead’ can be extracted from the DC responses and then used to anchor the values. One way to do this is by explicitly comparing the health state ‘dead’ to the EQ-5D-5L health states that are being judged in the DC task. An objection on theoretical grounds is that responses obtained from choices comparing heath states to dead may violate the random utility theory underlying the DC model. This happens when a subset of respondents consider all health states to be better than dead—for example, due to their religious beliefs. The size and effect of the bias are yet unknown; in practice, the bias may be small. Indeed, when this approach was adopted for the valuation of EQ-5D-3L health states [8], the results were promising. Whether or not this will also be so when it is used for EQ-5D-5L valuation will be expanded upon in this paper.

The primary objective of the study reported here was to examine the results of two different approaches to rescale DC models incorporating ‘dead’ into the utility scale as an anchor point and to compare the results with those obtained anchoring on lead-time TTO. A secondary objective was to evaluate the effect of excluding DC responses elicited from those who did not consider any health state to be worse than the health state dead.

## Methods

This pilot study used both a DC and a lead-time trade-off (lead-time TTO) approach to produce values for the set of 3,125 (5^5^) health states defined by the EQ-5D-5L instrument. As a detailed description of each approach in the context of health-state valuation can be found elsewhere [8, 13], only a brief summary will suffice here. The study design followed recommendations from the EuroQol Group Valuation Task Force and was part of a multi-country initiative to explore methodological uncertainties about the valuation protocol for a new EQ-5D-5L value set.

### Valuation of EQ-5D-5L health states

#### DC method

In the DC method, the respondents were asked to state their preference between two health states, A and B. This comparison of health states produces data that were subsequently analyzed to produce values on a latent scale. The profiles did not mention either their duration or what happens after these states. The DC design was generated using a Bayesian efficient approach [14] and consisted of 50 pairs of health states allocated to five blocks. These amounts were set in order to have sufficient power to estimate health-state values based on the proportions of choices between the pairs of states. To allow anchoring of the values on the ‘dead—full health’ scale, we extended the DC task by asking whether state A was worse than dead (WTD) and whether state B was WTD.

#### Lead-time TTO

The lead-time TTO method is an extension of the traditional TTO [13]. In a classic TTO, participants complete one task for health states considered better than dead and another task for those considered WTD. Lead-time TTO consists of a single task: to choose between Life A (*T* years in full health) and Life B [10 years in full health (lead time) plus 5 years in a target health state (disease time)]. All respondents start with Life A versus Life B where *T* = 15 years in 11111; depending on whether they choose A or B, the value of *T* is raised or lowered until the participants feel that A and B are the same. The lead-time TTO design was constructed with a Federov algorithm that allowed model parameters to be estimated without bias and with minimal variance [15]. The final lead-time TTO design contained 100 states in ten blocks.

### Data collection

Four hundred persons, who were representative of the Spanish population in terms of age, gender, and education, took part in this study. An online survey administered via the EuroQol Valuation Technology (EQ-VT) software was used to collect DC and lead-time TTO responses. The final survey included the EQ-5D-5L questionnaire, ten DC tasks, and five lead-time TTO tasks as well as demographic questions. Participants were also queried about the difficulty of the DC and lead-time TTO tasks and how well they had understood them. The EQ-VT randomly assigned each participant to a DC block and a lead-time TTO block. In both types of block, the tasks were presented in random order. Given the number of participants, the study yielded an average of 80 observations for each DC pair (400 participants × 10 states/50 pairs) and 20 observations for each lead-time TTO state (400 participants × 5 states/100 states).

A survey company administered the study in Barcelona (June 2011). The researchers JMRG, ME, MH, and JC supervised the data collection with assistance from the EuroQol Group. Participants were recruited using telephone directories for the metropolitan area of Barcelona, personal contacts, a database of panelists, or ‘snowballing’ from contacts of participants included in this study.

Eight groups, each with an average of ten respondents, were recruited per day during 6 days, yielding the target of 400 participants. Each participant was assigned a computer and given an ID number and a password. Two computer rooms were available for each session. Interviews were conducted by two trained interviewers and four members of the Spanish Valuation Team (JMRG, ME, MH, and JC).

### Statistical analysis

The sample as well as the DC and lead-time TTO responses were described with descriptive statistics. Four statistical models were used to estimate EQ-5D value sets: (1) a conditional logistic model, which produced the health-state values based only on choices between health states, thus ignoring responses to the dead questions (*N* = 397; henceforth DC_TTO_; (2) a rank-ordered logistic model, which was then used on the full DC dataset and included responses to the dead questions (*N* = 397, henceforth DC_dead_); (3) a rank-ordered logistic model, which used data only on those participants who chose at least one state worse than dead (*N* = 195, henceforth DC_WTD_); a linear regression model, which used the lead-time TTO responses (*N* = 373; henceforth called lead-time TTO). The three models that were estimated with DC responses had to be rescaled to indicate that 0 stands for dead and that 1 forms the upper bound for full health. This was achieved using the additional ‘dead’ questions in the DC experiments in the case of DC_dead_ and DC_WTD_. For the DC_TTO_ model, the worst health state predicted on the lead-time TTO model (profile 55555) was taken as an anchor point to rescale the arbitrary scale of the conditional logistic model. Details on each model are given below.

#### DC_TTO_ model

In the case of DC, the values are not directly observable and have to be calculated from the responses to the choice exercise. We assume that the participants choose the health state that gives them higher utility, so this can be modeled as a conditional logistic model. As such, the independent variable *Y*_*I*_ represents the choice of participant *I* between A or B. The model assumes a value decomposition in two parts, explainable by *V*_iA_ plus an error *ε*_*i*_. If errors are assumed to be random and to show a type 1 extreme value distribution, a conditional logistic model emerges [8, 16, 17]. Let us assume that component V of the value can be explained with an additive model:

1 $$ V_{\text{iA}} = \mathop \sum \limits_{j = 1}^{J} X_{\text{iAj}} \cdot \beta_{j} $$

where *X*_iAj_ are 20 dummies {0, 1}, per participant* i*, representing the severity levels for each dimension of EQ-5D-5L for state A. Then *β*_*j*_ will represent the coefficient for each independent variable* j*.

Accordingly, it is possible to estimate the coefficients of the model and thus to extrapolate values that have not been observed within the population by using the linear part of the DC_TTO_ model. The values obtained from the linear part of the model shown above are on an arbitrary scale. In order to rescale the values from the DC_TTO_ model, the extreme negative value estimated in the lead-time TTO model (55555) was used to anchor the DC_TTO_ 55555 health state to that value. Therefore, both models produce the same index value for the 55555 health state. To obtain a full set of utility decrements, every coefficient of the DC model is divided by the scalar (55555_lead-time TTO_ − 1)/(55555_DCTTO_ − 1). The outcome of this transformation for each coefficient yields the utility decrements for the DC_TTO_ model.

#### DC_dead_ model

A rank-order logistic analysis was performed for the DC_dead_ model [8]. In the same way as for a conditional logistic model, a two-part decomposition is assumed for the value. Where *V*_iA_, this model can be written as follows:

2 $$ V_{\text{iA}} = \mathop \sum \limits_{j = 1}^{20} X_{\text{iAj}} \cdot\beta_{j} + X_{{i   {\text{dead}}}} \cdot\beta_{\text{dead}} $$

Values are therefore obtained from the linear part (above) of the model on an arbitrary scale, as they are in the DC_TTO_ model. For this DC_dead_ model, the anchor point is the health state dead. Since the value for dead has to be 0, each coefficient is divided by $\beta_{\text{death}} .$ ensuring $\beta_{\text{death}}^{\prime }$ = −1. The final function to estimate index values is given by:

3 $$ V_{\text{iA}} = 1 - \mathop \sum \limits_{j = 1}^{20} X_{\text{iAj}} \cdot\beta^{'}_{j} + X_{{i {\text{dead}}}} \cdot\beta^{'}_{\text{dead}} $$

where $$ \beta_{j}^{\prime } = {\raise0.7ex\hbox{${\beta_{j} }$} \!\mathord{\left/ {\vphantom {{\beta_{j} } {abs(\beta_{\text{dead}} )}}}\right.\kern-0pt} \!\lower0.7ex\hbox{${abs(\beta_{\text{dead}} )}$}} $$.

#### DC_WTD_ model

The DC_WTD_ model was estimated as a rank-order logistic model similar to the DC_dead_ model. For this case, the data were restricted to responses from participants who chose at least one state worse than dead. This model was used to evaluate whether including participants who did not choose any state worse than dead would bias the coefficient estimates.

#### Lead-time TTO model

For lead-time TTO responses, a linear model was estimated. The specification of the model in its general form is:

4 $$ Y_{i} = \mathop \sum \limits_{j = 1}^{n} x_{ij} \cdot \beta_{j} + \varepsilon_{i} $$

where *Y*_*i*_ represents the observed values from lead-time TTO data for participant* i*. A continuous variable, which takes values between −2 and 1, was created. The lead-time TTO values *T* from the survey were transformed into a −2 and 1 scale using the formula (*T* − *T*_lead)/(*T*_total − *T*_lead). In our design, *T*_lead = 10 indicates that the additional years in full health occur at the beginning of the exercise, and *T*_total = 15 indicates the sum of T_lead and disease time (5 years). The independent variables *X*_*ij*_ are 20 dummies {0, 1} for each participant* i*, representing the severity levels for each dimension of EQ-5D-5L. *β*_*j*_ represents the coefficients for each independent variable* j*;* ε*_*i*_ represents the errors for each participant *i*. Different specifications used in previously published examples were explored in order to fit the best model [2–5]. However, none of the models led to improved goodness of fit measured with log-likelihood, nor did they correct any inconsistencies in the models’ coefficients. Therefore, the lead-time TTO model presented in this study was estimated using a simple ordinary least squares model. Finally, a function to estimate values for each health state was created using the regression model specified in the following equation:

5 $$ Y_{i} = 1 - (\beta_{0} + \beta_{1} \cdot {\text{mo}}2_{i} + \beta_{2} \cdot {\text{mo}}3_{i} + \beta_{3} \cdot {\text{mo}}4_{i} + \beta_{4} \cdot {\text{mo}}5_{i} + \cdots + \beta_{20} \cdot {\text{ad}}5_{i} + \varepsilon_{i} ) $$

with mo2, mo3, mo4, mo5, sc2, sc3…, ad4, and ad5 indicating the corresponding dummy for the EQ-5D-5L severity level.

To compare the four models, we used descriptive statistics and quantile–quantile plots (Q-Q plots) of the value sets obtained from the different models. A Q-Q plot sets off estimates of the quantiles of two distributions against each other, and the pattern of points it displays is used to compare the two distributions of value sets. In addition, the value sets produced for each model are compared using the mean square difference (MSD) and concordance correlation coefficient (CCC) [18]. All values for the 3,125 health states are estimated by each of the estimated models. For each one:one comparison (model 1 vs. model 2), the MSD is calculated as follows:

6 $$ {\text{MSD}}_{{\bmod {\text{el}}1 {\text{vs}} \bmod {\text{el}}2}} = \frac{{\mathop \sum \nolimits_{i = 1}^{3,125} ({\text{indexvalue}}_{{\bmod {\text{el}}1_{i} }} - {\text{indexvalue}}_{{\bmod {\text{el}}2_{i} }} )^{2} }}{3,125} $$

All statistical analyses were performed on STATA 11 MP (StataCorp LP, College Station, TX).

## Results

### Sample characteristics

The study cohort comprised 400 persons with a mean age (standard deviation, SD) of 44.1 (16.9) years; and 59.7 % (239) were male (Table 1). More than half were employed or freelance and 15 % were retired. Less than half (43.75 %; 175) were in full health (11111). Few reported extreme or severe problems in any dimension of the EQ-5D-5L (three was the maximum number of respondents reporting extreme problems in the ‘usual activities’ dimension; see Table 2).

**Table 1:** Descriptive statistics of study sample (*N* = 400)

Characteristics	Value^a^
Age (mean ± SD)	44.1 ± 16.9
Gender	
Male	239 (59.7)
Female	161 (41.3)
Employment status	
Domestic tasks	13 (3.25)
Employed or freelance	201 (50.25)
Student	39 (9.75)
Retired	59 (14.75)
Unemployed	60 (15)
Data missing	28 (7)
Education	
Higher education	110 (27.5)
High school	175 (43.75)
Primary school	86 (21.5)
Data missing	29 (7.25)
Experience severe illness	
Self	63 (15.75)
Relatives	278 (69.5)
Other	136 (34)

*SD* standard deviation

^a^Data are presented as the number (*N*) of subjects with the percentage of total subject cohort given in parenthesis, unless stated otherwise

**Table 2:** Distribution of EQ-5D-5L responses across participants

Level of response	Mobility	Self care	Usual activities	Pain/discomfort	Anxiety/depression
No problems	337 (84.9)	383 (96.5)	352 (88.7)	239 (60.2)	271 (68.3)
Slight problems	35 (8.8)	8 (2)	31 (7.8)	119 (30)	95 (23.9)
Moderate problems	21 (5.3)	5 (1.3)	10 (2.5)	30 (7.6)	22 (5.5)
Severe problems	3 (0.8)	0 (0)	1 (0.3)	8 (2)	9 (2.3)
Unable/extreme	1 (0.3)	1 (0.3)	3 (0.8)	1 (0.3)	0 (0.0)

Data are presented as the number (*N*) of subject cohort with the percentage given in parenthesis

### Descriptive statistics

The DC responses were 61.7 % for state A and 38.3 % for state B. Reflecting differences in the impact of dimensions and levels on health status, not all choices followed the misery index (sum of the levels across domains) order. For example, the observed probability for choosing state 55534 over state 33355 was 0.852. Only 2.4 % of all respondents thought that state 55534 was WTD and 14.81 % thought that 33355 was WTD (Table 3). Some inconsistencies were observed in the estimated lead-time TTO valuations. For example, health state 55253 had a lower mean value (−0.4) than health state 55255 (−0.147) (*P* = 0.0004), even though the latter clearly dominates in term of severity of the five health domains (Table 4). A total of 195 (48.75 %) participants using DC and 216 (54 %) using lead-time TTO rated at least one state as WTD.

**Table 3:** Discrete choice responses for the 50 paired scenarios included in the valuation exercise

Profile A (misery index)	Profile B (misery index)	A (%)	WTD (%) A	WTD (%) B	Profile A (misery index)	Profile B (misery index)	A (%)	WTD (%) A	WTD (%) B
11445 (15)	32115 (12)	58.02	2.47	8.64	33223 (13)	21232 (10)	85.54	2.41	7.23
13334 (14)	45441 (18)	19.75	3.70	13.58	33432 (15)	15551 (17)	37.04	2.47	6.17
14122 (10)	54231 (15)	55.42	6.02	25.30	34134 (15)	45325 (19)	93.83	2.47	7.41
14533 (16)	21542 (14)	24.69	3.70	13.58	34255 (19)	35221 (13)	44.74	2.63	9.21
14552 (17)	55325 (20)	93.83	7.41	40.74	35235 (18)	42325 (16)	10.53	0.00	15.79
15351 (15)	14312 (11)	51.32	2.63	14.47	35252 (17)	32254 (16)	33.33	7.41	18.52
15555 (21)	53455 (22)	78.31	6.02	24.10	35312 (14)	14422 (13)	74.36	2.56	20.51
21235 (13)	12243 (12)	24.69	2.47	8.64	41114 (11)	24142 (13)	98.72	3.85	37.18
21445 (16)	55141 (16)	24.36	2.56	24.36	41312 (11)	24253 (16)	37.04	2.47	16.05
21522 (12)	25324 (16)	62.96	9.88	24.69	42122 (11)	31325 (14)	88.46	1.28	10.26
22341 (12)	45145 (19)	74.36	2.56	20.51	42153 (15)	53151 (15)	96.15	1.28	17.95
22544 (17)	35452 (19)	85.19	4.94	16.05	42255 (18)	55524 (21)	48.68	3.95	13.16
23122 (10)	12415 (13)	18.42	1.32	5.26	42441 (15)	21415 (13)	71.08	4.82	12.05
23134 (13)	14314 (13)	85.53	6.58	17.11	43245 (18)	34324 (16)	61.73	2.47	6.17
23231 (11)	25323 (15)	70.37	3.70	27.16	43412 (14)	13342 (13)	51.81	8.43	15.66
23442 (15)	25414 (16)	83.95	3.70	19.75	43514 (17)	23321 (11)	83.33	0.00	6.41
23451 (15)	34354 (19)	79.01	6.17	30.86	44115 (15)	21455 (17)	32.53	9.64	39.76
24453 (18)	41331 (12)	87.65	2.47	30.86	44151 (15)	53242 (16)	75.00	6.58	17.11
25235 (17)	13413 (12)	83.95	2.47	13.58	44234 (17)	33441 (15)	60.24	3.61	21.69
31451 (14)	45431 (17)	80.72	4.82	10.84	45515 (20)	34433 (17)	14.10	5.13	24.36
31452 (15)	13141 (10)	37.04	12.35	32.10	51331 (13)	22421 (11)	85.90	7.69	23.08
31521 (12)	43152 (15)	84.21	0.00	18.42	51552 (18)	35513 (17)	13.25	0.00	7.23
32211 (9)	14211 (9)	88.89	1.23	12.35	54121 (13)	44322 (15)	80.77	1.28	12.82
32241 (12)	51525 (18)	40.79	3.95	17.11	54424 (19)	15321 (12)	67.11	1.32	9.21
33111 (9)	32545 (19)	61.45	10.84	19.28	55534 (22)	33355 (19)	85.19	2.47	14.81

*WTD* heath state assessment of 'worse than dead'

**Table 4:** Mean lead-time trade-off values and percentage of values WTD for the health states included in the valuation exercise

Profile	Value	Std error	WTD (%)	Profile	Value	Std error	WTD (%)	Profile	Value	Std error	WTD (%)	Profile	Value	Std error	WTD (%)
11112	0.786	0.323	4.76	14335	0.041	0.852	18.18	25555	−0.184	0.978	31.82	44415	−0.068	0.700	36.84
11114	0.363	0.614	10.53	14411	−0.006	0.887	33.33	33133	0.483	0.746	9.52	52221	0.503	0.813	11.76
11115	0.075	0.667	27.78	14413	0.081	0.913	33.33	33331	0.263	0.809	10.53	52225	0.379	0.567	19.05
11121	0.629	0.630	10.53	14415	0.264	0.703	11.11	33333	0.470	0.641	10.00	52251	−0.061	0.933	22.73
11122	0.456	0.739	16.67	14441	−0.277	0.920	40.91	33334	0.471	0.365	10.53	52255	−0.038	0.920	33.33
11141	0.335	0.887	17.65	21111	0.664	0.439	0.00	33345	0.008	0.651	25.00	52324	0.161	0.603	31.58
11144	−0.087	0.719	21.05	21112	0.505	0.647	14.29	35251	−0.129	0.790	38.10	52521	−0.216	0.920	47.37
11145	0.274	0.686	33.33	21115	0.326	0.656	23.81	35525	−0.035	0.929	35.00	52525	0.081	0.901	28.57
11211	0.562	0.781	9.52	22251	−0.050	0.998	37.50	41111	0.635	0.492	5.00	52551	−0.608	1.010	65.00
11212	0.422	0.623	8.70	22521	0.224	0.838	26.09	41115	−0.009	0.906	36.36	52555	−0.406	0.826	50.00
11221	0.534	0.572	9.09	22525	0.183	0.815	17.39	41141	0.161	0.566	26.32	53251	0.150	0.630	33.33
11245	−0.053	0.799	38.89	22551	0.036	0.728	16.67	41143	0.266	0.695	21.05	53521	0.093	0.923	22.73
11411	0.571	0.561	14.29	22553	0.253	0.654	16.67	41145	−0.075	0.733	33.33	53555	−0.337	0.964	47.37
11413	0.447	0.746	5.88	22555	−0.463	0.887	56.25	41343	−0.100	0.823	30.00	55221	0.329	0.605	10.53
11415	0.119	0.860	33.33	23255	−0.187	0.623	31.58	41411	0.421	0.365	5.26	55225	−0.197	0.838	44.44
11441	0.075	0.905	35.00	25221	−0.053	0.972	31.25	41413	0.032	0.863	31.82	55235	0.003	0.942	35.00
11445	−0.134	0.778	42.11	25225	−0.113	0.898	40.00	41415	−0.175	0.921	31.25	55251	−0.287	0.950	52.17
12111	0.681	0.536	11.11	25251	−0.110	0.785	42.86	41441	0.184	0.505	26.32	55253	−0.400	1.062	44.44
12112	0.624	0.525	5.26	25255	0.105	0.595	38.10	41445	0.286	0.736	11.11	55255	−0.147	0.888	41.18
14111	0.266	0.536	15.79	25455	−0.053	0.763	31.58	44111	0.059	0.870	31.25	55521	0.167	0.651	23.81
14113	0.328	0.808	15.00	25521	0.389	0.374	5.26	44113	0.256	0.683	11.11	55523	−0.114	0.772	47.62
14115	0.308	0.650	15.79	25525	0.097	0.937	23.53	44115	−0.289	0.987	50.00	55525	−0.337	0.810	42.11
14141	−0.130	0.907	36.36	25531	0.189	0.694	26.09	44141	0.233	0.582	22.22	55551	−0.289	0.857	44.44
14143	0.002	0.903	40.00	25551	0.074	0.676	23.81	44145	−0.215	1.027	39.13	55553	−0.329	0.909	52.38
14145	0.050	0.703	31.58	25553	0.026	0.795	36.84	44411	0.125	0.645	25.00	55555	−0.545	0.935	52.63

* Std error* standard error

### Models

For the estimation of the three DC models, we omitted two respondents from the analysis because their DC choices were always A or always B; the 328 responses without a logical order among state A, state B, and dead were also omitted. For the lead-time TTO model, it was necessary to clean the dataset for inconsistencies. In this case 24 respondents with the same value for all TTO tasks were excluded from the analysis, as were two respondents for whom data were missing due to technical problems.

Several model specifications were explored. However, only main effects models are presented here. The others did not perform better in terms of having fewer inconsistencies or maximizing the likelihood function. In order to allow comparison among the models’ coefficients, we present here the rescaled coefficients for the three final DC models. The DC_WTD_ model has the highest likelihood value (−1,401.549), but DC_TTO_ performs better than DC_dead_ (−1,791.37 vs. −2,700.25 respectively) (Table 5).

**Table 5:** Parameter estimates for the models^a^ based on data derived by discrete choice and lead-time trade-off values

Dummy	DC_dead_ model, *N* = 397, observation = 21,852			DC_WTD_ model *N* = 195, observation = 9,726			DC_TTO_ model, *N* = 397, observation = 7,940			Lead-time TTO model, *N* = 373, observation = 1,864			DC_dead_ rescaled	DC_WTD_ rescaled	DC rescaled
	Coefficient	Std. error	*P* > *z*	Coefficient	Std. error	*P* > *z*	Coefficient	Std. error	*P* > *z*	Coefficient	Std. error	*P* > *t*	Coefficient	Coefficient	Coefficient
MO2	−0.365	0.098	0.00	−0.418	0.141	0.00	−0.449	0.108	0.00	−0.042	0.092	0.652	−0.056	−0.109	−0.106
MO3	−0.370	0.093	0.00	−0.446	0.134	0.00	−0.408	0.105	0.00	−0.091	0.131	0.489	−0.057	−0.111	−0.096
MO4	−1.021	0.106	0.00	−1.150	0.154	0.00	−1.115	0.12	0.00	−0.128	0.056	0.022	−0.157	−0.266	−0.264
MO5	−1.445	0.108	0.00	−1.470	0.154	0.00	−1.596	0.127	0.00	−0.251	0.102	0.014	−0.223	−0.331	−0.378
SC2	−0.292	0.091	0.00	−0.298	0.134	0.03	−0.239	0.102	0.02	0.098	0.098	0.319	−0.045	−0.061	−0.057
SC3	−0.273	0.088	0.00	−0.288	0.128	0.02	−0.224	0.098	0.02	0.133	0.123	0.280	−0.042	−0.054	−0.053
SC4	−1.018	0.108	0.00	−1.016	0.153	0.00	−1.118	0.124	0.00	−0.256	0.056	0.000	−0.157	−0.26	−0.265
SC5	−1.041	0.081	0.00	−0.922	0.117	0.00	−1.132	0.089	0.00	−0.042	0.106	0.693	−0.160	−0.226	−0.268
UA2	−0.428	0.085	0.00	−0.488	0.125	0.00	−0.51	0.095	0.00	−0.111	0.08	0.165	−0.066	−0.103	−0.121
UA3	−0.475	0.088	0.00	−0.562	0.126	0.00	−0.479	0.099	0.00	−0.175	0.099	0.076	−0.073	−0.124	−0.113
UA4	−0.781	0.084	0.00	−0.812	0.128	0.00	−0.839	0.092	0.00	−0.124	0.057	0.030	−0.120	−0.145	−0.198
UA5	−0.872	0.094	0.00	−0.757	0.134	0.00	−0.953	0.11	0.00	−0.267	0.087	0.002	−0.134	−0.176	−0.225
PD2	−0.098	0.093	0.29	−0.245	0.137	0.07	−0.034	0.104	0.74	−0.021	0.086	0.811	−0.015	−0.028	−0.008
PD3	0.004	0.097	0.97	−0.115	0.145	0.43	0.091	0.109	0.40	0.036	0.105	0.730	0.001	0.001	0.022
PD4	−0.922	0.107	0.00	−1.003	0.156	0.00	−0.893	0.121	0.00	−0.238	0.056	0.000	−0.142	−0.217	−0.211
PD5	−1.213	0.112	0.00	−1.057	0.157	0.00	−1.441	0.133	0.00	−0.348	0.093	0.000	−0.187	−0.275	−0.341
AD2	−0.398	0.095	0.00	−0.332	0.139	0.02	−0.412	0.104	0.00	0.055	0.092	0.553	−0.061	−0.082	−0.098
AD3	−0.760	0.103	0.00	−0.621	0.143	0.00	−0.819	0.12	0.00	−0.015	0.056	0.790	−0.117	−0.164	−0.194
AD4	−1.079	0.108	0.00	−0.961	0.152	0.00	−1.146	0.126	0.00	−0.161	0.105	0.125	−0.166	−0.246	−0.271
AD5	−1.271	0.103	0.00	−1.164	0.142	0.00	−1.369	0.12	0.00	−0.176	0.041	0.000	−0.196	−0.278	−0.324
Intercept	NA	NA	NA	NA	NA	NA	NA	NA	NA	−0.452	0.066	0.000	NA	NA	NA
DEAD	−6.494	0.187	0.00	−5.346	0.262	0.00	NA	NA	NA	NA	NA	NA	NA	NA	NA
							Log *L* = −1,791.3742								
	Log *L* = −2,700.2528			Log *L* = −1,401.5487						*R* ^2^ = 0.1066					
							Pseudo *R* ^2^ = 0.2202								

*DC* Discrete choice; NA, data not available/analyzed

^a^For a full description of each model, see section "Statistics"

Regarding the rescaling method for DC models, the value for 55555 was estimated with a lead-time TTO model to be −0.535. This value was used to anchor the DC_TTO_ model, which previously had a value of −5.491 for state 55555. The ratio to rescale the coefficients was abs [(−5.491 − 1)/(−0.535 − 1)] = 4.228. The final rescaled coefficients for DC_TTO_ are *β*′_*j*_ = *β*_*j*_/4.228. In DC_dead_ models, the dead state has a value of 0. The coefficient for the dead state β_dead_ in the DC_dead_ model is −6.494, since this coefficient must be −1 (meaning that the dead state has a value of 0). The rescaled coefficients are then *β*′_*j*_ = *β*_*j*_/6.494. If the coefficient for the dead state β_dead_ in the DC_WTD_ model is −5.346, then the rescaled coefficients are *β*′_*j*_ = *β*_*j*_ */*5.346.

In general, values in the lead-time TTO model were lower than in any of the DC rescaled models due to the estimated intercept value of 0.452. However, there are several inconsistencies for some estimated coefficients. In all of the estimated models, for example, the coefficient for moderate problems (level 3) in the pain/discomfort domain is positive, although not statistically significant. Other inconsistencies are statistically significant: the lower coefficients for slight (level 2) compared to moderate problems (level 3) in the self-care domain for the three DC models and in the mobility and usual-activities domain for DC. The value of the 55555 state in the DC_dead_ model (0.100) was higher than the corresponding value for the DC_WTD_ model (−0.004); however, for both DC_dead_ models, these values were much higher than that in the lead-time TTO model (−0.535).

**Fig. 1:**
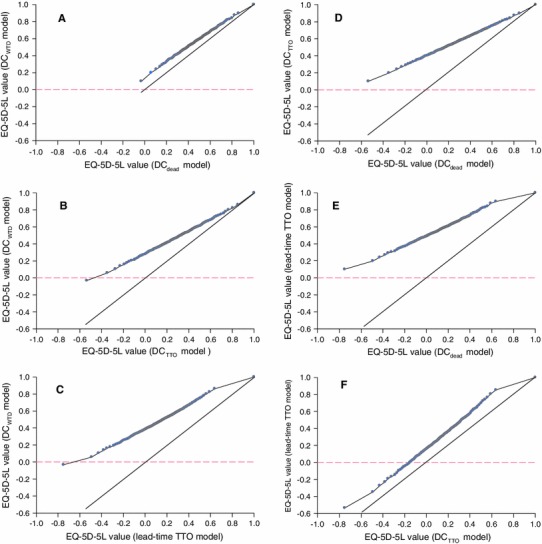
Quantile-quantile plots for comparison of values obtained from DC_dead_, DC_WTD_, DC_TTO_, and lead-time trade-off (*TTO*) models. For a full description of each model, see section "Statistics"

The two DC dead models are in concordance, with DC_dead_ versus DC_WTD_ having CCC = 0.848, and DC_TTO_ versus lead-time TTO having CCC = 0.725 as well. However, the concordance among the remaing models is lower: (1) DC_WTD_ vs. DC_TTO_ : CCC: 0.677; (2) DC_dead_ versus DC_TTO_: CCC = 0.478; (3) DC_dead_ versus lead-time TTO: CCC = 0.239; (4) DC_WTD_ vs. lead-time TTO: CCC = 0.349. Compared to DC models, lead-time TTO produced lower values for practically every health state (Fig. 1c, e, f). Both DC_dead_ and DC_WTD_ models estimated very similar values (Fig. 1a).

The MSD for differences between the 3,125 states in both DC_dead_ models is 0.009. However, the MSD for the differences with the lead-time TTO model are 0.217, 0.142, and 0.045 for the DC_dead_, DC_WTD_, and DC_TTO_ models, respectively. The MSD for the differences with DC_TTO_ are 0.091 and 0.044 for DC_dead_ and DC_WTD_, respectively.

## Discussion and conclusions

In the study reported here we compared two approaches for rescaling DC values on the dead (0)—full health (0) scale to obtain an EQ-5D-5L value set that can be used in economic evaluation. The two approaches were: (1) DC incorporating an additional judgmental task in which the health state ‘dead’ is assessed against other health states; and (2) a DC model anchoring on lead-time TTO values.

None of the estimated models were completely consistent in terms of regression coefficients. All models had some positive coefficients. Also, to be consistent, a model must meet the condition that each dimension should satisfy an increasing order in the absolute value of the coefficients for each level of severity. According to the results, each of the models did satisfy the condition for some dimensions—but not for all. The DC_TTO_ model did not satisfy the condition more often than the DC_dead_ models, and its rescaled results produced higher utility decrements than both rescaled DC_dead_ models. The rescaled DC_WTD_ model differs less from rescaled DC_TTO_ than from rescaled DC_dead_. However, we have to take into account that the intercept for the lead-time TTO model was extremely high, which leads to health state values that lack face validity. For example, a person with slight mobility problems has a value of <0.55, which is ridiculous when compared to the previous EQ-5D value set [2–5].

The reason for the inconsistencies in the logistic regression results is not clear. On the one hand, these inconsistencies could be explained by the fact that the DC design included only 50 pairs of health states, which may be inadequate to yield sufficient information (and thus power) to estimate the logistic models (some coefficients were not statistically significant). On the other hand, more power (thus, a larger sample size) may be needed for each pair of health states when the number of pairs is fixed. When the data were applied to the Spanish arm of the multi-country study, the inconsistencies in the DC model disappeared [19]; however that study had both more pairs (200) and more observations per pair. The questions touching upon dead, which are necessary for the DC_dead_ models, were only conducted in the Spanish pilot study. Therefore, the analysis of DC_dead_ models could not be extended to all countries for the sake of comparison. In that light, it would make sense to increase the number of pairs in the DC design that touch upon dead and also to increase the power per pair as this approach would ensure that future studies conducted by using a DC model incorporating dead will be consistent for the whole multi-country dataset.

On comparing the results of the modeling exercise for all participants versus those who rated at least one state as WTD, we found that the DC_dead_ and DC_WTD_ models produced similar results, with the only difference being the position of ‘dead’. In particular, we found higher utility decrements and thus lower health state values for EQ-5D-5L states when the participants who did not rate any state as WTD were removed from the analysis. However, this may not amount to bias and may simply reflect the preferences of the population. Whatever the reason, the impact on actual results was not large. It should be kept in mind that this was not a direct comparison, as the participants it covered were not identical. From a mathematical point of view and based on the RUT theory, estimation may fail when many participants do not choose any WTD option. Nevertheless, the DC_dead_ model could be estimated and did not perform much worse than the DC_WTD_ model in terms of likelihood.

There is some concern about the feasibility of some elements of the DC and lead-time TTO as conducted in this survey. In general, the participants understood the hypothetical nature of the health states and lives they were presented with. They knew they had to choose the health state/life that they preferred rather than the health state/life with which they identified the most. However, some problems arose in the course of both exercises, especially during the lead-time TTO task. Many individuals were confused when making choices and did not realize that the health conditions changed when they answered that ‘both lives are almost equal’. Although this consequence had been explained, it was necessary for the administrators to do the first lead-time TTO exercise together with the participants so they could do the rest of the exercises as required. The general impression was that many of the respondents did not answer the TTO part of the exercises appropriately. Some individuals reported that they could not decide when they were indifferent between both lives because they always preferred Life B. This indecisiveness could explain the illogical results obtained with the lead-time TTO model. In general, the respondents needed less assistance on the DC part of the survey, but many did comment on the difficulty of making choices between health states. The difficulties they encountered in the survey tasks emphasize the important role of the face-to-face interviews that are also part of the study design. DC and lead-time TTO elicitation techniques require the respondents to compare health states with ‘dead’; this question was posed directly in each of the DC exercises and indirectly in each of the lead-time TTO exercises. From the results we can deduce that a state was more frequently considered WTD in indirect (lead-time TTO) than direct questions (DC + dead), possibly due to the fact that in lead-time TTO the distinction between negative and positive values was not explicitly made. This fact could explain the lower values observed for the lead-time TTO method and hence the DC_TTO_.

Previous studies have investigated the incorporation of the health state dead in the DC task [8, 16, 17]. However, none of these used the EQ-5D-5L to allow a direct comparison. Stolk et al. [8] used the classic three-level version of EQ-5D. Our results do not confirm those obtained by Stolk et al., probably because their comparison was made with classic instead of lead-time TTO. Also, the five-level version makes the DC task more complicated for the respondents, and this complexity might have led some participants to make random choices when they could not decide between health states A and B.

DC_dead_ models produce correlated results with slight differences (no bias). Incorporating the health state dead into the general DC technique produces results in concordance with the DC_TTO_. DC modeling warrants further research to optimize the design if it is to be used to estimate EQ-5D-5L value sets. The lead-time TTO produces very high utility decrements, and its consistency among responses is lower than that of DC models.
